# Early osteoimmunomodulatory effects of magnesium–calcium–zinc alloys

**DOI:** 10.1177/20417314211047100

**Published:** 2021-09-22

**Authors:** Maryam Rahmati, Sabine Stötzel, Thaqif El Khassawna, Kamila Iskhahova, DC Florian Wieland, Berit Zeller Plumhoff, Håvard Jostein Haugen

**Affiliations:** 1 Department of Biomaterials, Institute for Clinical Dentistry, University of Oslo, Oslo, Norway; 2 Experimental Trauma Surgery, Justus-Liebig University Giessen, Giessen, Germany; 3 Faculty of Health Sciences, University of Applied Sciences, Giessen, Germany; 4 Institute of Metallic Biomaterials, Helmholtz-Zentrum Hereon, Geesthacht, Germany

**Keywords:** Magnesium, zinc, calcium, macrophage polarization, biomineralization

## Abstract

Today, substantial attention is given to biomaterial strategies for bone regeneration, and among them, there is a growing interest in using immunomodulatory biomaterials. The ability of a biomaterial to induce neo vascularization and macrophage polarization is a major factor in defining its success. Magnesium (Mg)-based degradable alloys have attracted significant attention for bone regeneration owing to their biodegradability and potential for avoiding secondary removal surgeries. However, there is insufficient evidence in the literature regarding the early inflammatory responses to these alloys in vivo. In this study, we investigated the early body responses to Mg-0.45wt%Zn-0.45wt%Ca pin-shaped alloy (known as ZX00 alloy) in rat femora 2, 5, and 10 days after implantation. We used 3D micro computed tomography (µCT), histological, immunohistochemical, histomorphometrical, and small angle X-ray scattering (SAXS) analyses to study new bone formation, early macrophage polarization, neo vascularization, and bone quality at the implant bone interface. The expression of macrophage type 2 biological markers increased significantly after 10 days of Mg alloy implantation, indicating its potential in stimulating macrophage polarization. Our biomineralization results using µCT as well as histological stained sections did not indicate any statistically significant differences between different time points for both groups. The activity of alkaline phosphatase (ALP) and Runt-related transcription factor 2 (Runx 2) biological markers decreased significantly for Mg group, indicating less osteoblast activity. Generally, our results supported the potential of ZX00 alloy to enhance the expression of macrophage polarization in vivo; however, we could not observe any statistically significant changes regarding biomineralization.

## Introduction

Bone fracture is a major cause of severe physical disability and global socio-economic burden.^1–3^ Over the past few decades, biomaterials and interface tissue engineering fields have made considerable progress in suggesting promising strategies to stimulate tissue regeneration after bone tissue damage and/or loss caused by trauma, pathology, and resorption.^4–6^ Traditionally, titanium alloys (known as bio-inert metallic implants) are considered the gold standard for stabilizing bone fractures.^7^ Compared to other implants such as stainless steel, titanium alloys are developed as promising bone implants due to their good biocompatibility, corrosion resistance, and closer modulus to bone.^8–12^ However, due to some limitations (such as stress shielding and secondary operations for implant removal), bio-inert non-degradable implants could not be an optimal choice for bone regeneration.^13–15^ A perfect internal fixation device should degrade and reduce its stiffness over time as the fracture heals and does not need secondary operations for implant removal.^15^

On the other hand, the homeostasis of the immune system state is crucial for tissue healing.^16^ The tissue injury caused by indwelling biomaterials induces several chemical signaling cascades, resulting in a sequence of acute and chronic inflammatory responses leading to wound healing.^17–19^ Protein adsorption, neutrophils, and type 1 macrophages direct the expression of pro-inflammatory markers, which are in charge for provisional matrix development and wound site cleaning. This phase can take from hours to several days.^20^ After releasing biochemical cues, blood vessels expand and stimulate more blood flows into the injured area. In a normal wound healing process, the released type 1 macrophages polarize to type 2 macrophages, which mainly direct the expression of anti-inflammatory markers for about 2 weeks. During this phase, tissue granulation, fibroblast infiltration, neo-vascularization, and consequently wound healing occur.^18,20^ After the biomaterial implantation, the time point of macrophage polarization from type 1 to 2 is affected by the injury caused by surgical operations, tissue type, and biomaterial physicochemical properties.^17,20^ The ability of a biomaterial to stimulate neo-vascularization and macrophage polarization defines its success. Reducing the host responses through modulating macrophage polarization has been the focus of many recent studies.^18,21–24^ In addition, the monocyte-macrophage cell lineage is known as a key player in bone regeneration and acute inflammatory response. This is largely owing to their high plasticity in response to environmental signals and their multiple roles in bone homeostasis.^25^

Over the past few years, biomedical scientists and engineers have developed biodegradable metallic materials to improve the regenerative capability of biomaterials and stimulate the desirable immune system responses leading to the biomaterial’s success.^23,26–28^ Such materials are developed to adjust their function in the body based on the biochemical and biomechanical properties of bone tissue.^29^ A biodegradable biomaterial acts as a support for the surrounding cells/tissue to grow in, and therefore guides the healing processes toward new bone formation.^30,31^ After repairing the damaged tissues, the implant is removed through in vivo degradation to non-toxic products, which reduces the need for a second surgical event to remove the implant.^13–15^

Among different degradable materials, magnesium (Mg) implants have gained substantial attention as a superior alternative material for bio-inert implants to induce less inflammatory responses and better bone formation.^21,23,32,33^ Mg-based implants overcome the stress shielding issues of titanium-based and stainless steel implants and the low mechanical stability of polymers.^21^ The density of Mg-based implants (1.7–2.0 g/cm^3^) is closer to that of bone (1.8–2.1 g/cm^3^) compared to titanium alloys (4.42 g/cm^3^ for Ti-6Al-4V), stainless steels (about 7.8 g/cm^3^), biodegradable poly(L-lactide) (about 1 g/cm^3^), and hydroxyapatite (3.156 g/cm^3^). In addition, the elastic modulus of Mg-based implants (~45 GPA) is closer to that of bone compared to titanium alloys and stainless steels with a modulus of about 110 and 200 GPA, respectively.^34–36^ Therefore, the stress shielding effect made by the high mismatch in elastic modulus and density between the native bone and implants should be diminished after Mg implantation.^36^ Mg is the second most abundant cellular cation and is a key player in regulating the immune system responses.^21^ Over the last decade, many studies reported the favorable functional properties of Mg-based implants toward bone regeneration.^37–39^ Zhang et al.^32^ demonstrated that magnesium implants stimulate bone formation through increasing the expression of Calcitonin Gene-Related Peptide (CGRP), as a neuropeptide released from the periosteum.^32^ Reifenrath et al.^33^ compared the osteoinductive properties and tissue responses of pure Mg with bio-inert titanium and degradable glyconate implants in mice after 2, 4, 8, 16, and 32 weeks of healing.^33^ They observed less host body reactions in Mg compared to other groups over time.^33^ In addition, Cheng et al.^40^ demonstrated that Mg porous scaffolds could reduce the inflammatory responses as well as stimulate the expression of collagen type 1 and osteopontin markers leading to enhanced bone formation after 1, 3, 5, 7, 10, and 14 days in vitro cell culture and 8 weeks in vivo implantation.^40^ The pro-inflammatory cytokines caused by magnesium ion’s deficiency could lead to osteoclastogenesis,^41,42^ whereas the magnesium-induced anti-inflammatory cytokines and tissue repair factors benefit tissue healing.^43,44^ The encapsulation of magnesium into titanium and calcium phosphate cement enhanced the macrophage type 2 polarization.^21,45^

However, the fast degradation rate and hydrogen gas formation of Mg could potentially affect the host responses in vivo. More recent studies focus on overcoming these issues by modifying its physicochemical properties with surface coating and/or alloy development strategies.^46,47^ Biodegradable alloys made of Mg, zinc (Zn), and calcium (Ca), known as Mg-Zn-Ca ternary or ZX alloys, are among the most recent developed Mg-based alloys that have gained attention in the field.^7,48–51^ Theoretically, due to the presence of all three elements in the body, the organism could metabolize the implanted alloy safely.^52^ Cipriano et al.^53^ studied the influence of degradation products over time on bone cell functions and reported that the ZX alloys can increase the cell functions toward new bone formation.^30^ However, as above-mentioned, immune cells, especially macrophages also play key roles in directing the host responses to biomaterials and we should study their responses to ZX alloys. To address this point, Costantino et al.^23^ studied the in vitro effects of Mg-based alloys and their degradation products on macrophage polarization, and observed that the alloys could stimulate the expression of both pro- and anti-inflammatory factors. Although it is undeniable that the biomaterial’s behavior in the body could be different to the in vitro conditions, in vivo studies on the early inflammatory and bone tissue responses to Mg-based alloys are still lacking in the literature.

Hence, this study aimed to examine the early blood vessel, macrophages, and bone cell responses 2, 5, and 10 days, after implantation. Therefore, 12 ZX00 pin-shaped implants (Mg-0.45wt%Zn-0.45wt%Ca) were inserted in the metaphysis of 12 rat femurs. Nonetheless, to examine the healing process without initial defect gap filling with implants, an empty defect group was examined as a control group. However, a direct compression of tissue properties between the two defect types with and without materials is impossible. Therefore, the groups were examined without statistical correlation. Whereas, the healing progression between the time points was compared in all groups with and without materials. Our null hypothesis was that the Mg-based implant could stimulate macrophage polarization and osteogenesis in vivo. Different 2 and 3D imaging technologies have been recently developed to study the bone-biomaterial interface in the body.^54–57^ In this study, we used different histology, immunohistochemistry, histomorphometry, 3D micro computed tomography (µCT), and small-angle X-ray scattering (SAXS) analyses to evaluate the tissue responses to the Mg alloys. We hypothesized that the results obtained from both 2 and 3D imaging technologies would be comparable.

## Materials and methods

### Materials development

The purified Mg was alloyed with zinc and calcium to synthesize the Mg-0.45wt%Zn-0.45wt%Ca pin-shaped alloy with a diameter of 1.6 mm and length of 8 mm (known as ZX00 alloy). Readers could find the details of alloy development by Grün et al.^50^

### Animal surgery

The animal experiments were performed at the Department of Orthopedics and Traumatology, Medical University of Graz. All animal experiments were done under the animal ethical respect, complied with the ARRIVE guidelines, and were authorized by the Austrian Ministry of Science and Research (accreditation number BMBWF-66.010/0066-V/3b/2019). We purchased 4-week-old female Sprague-Dawley^®^ (SD) rats (n = 12) from Janvier Laboratories (Saint Berthevin, France), and kept them on normal feed during the study. Rats were housed in groups of four in clear plastic cages on standard bedding. Water and a standard pellet diet were given ad libitum. At 6 weeks of age, the animals were divided into three groups to study the host responses 2, 5, and 10 days after implantation. Mg pin-shaped alloys (Mg-0.45wt%Zn-0.45wt%Ca) with a diameter of 1.6 mm were transcortically implanted into the right femur. A defect was created in the left leg, thereby serving as the sham control (the diameter of the drill hole was 1.6 mm). Readers could find the details of surgical and post-operative procedures in the Kraus et al.^58^ paper. Generally, after the specified time points, the explanted bone tissues were dissected from soft tissues, fixed in phosphate-buffered 4% paraformaldehyde, and kept at 4°C for 2 days.

### 3D micro-CT (µCT) analysis

Samples were scanned with a commercially available desktop µCT scanner (Skyscan 1172, Bruker micro-CT, Kontich, Belgium). This system (Skyscan 1172) contains an X-ray µfocus tube of 5 μm spot size with a high-voltage power supply, a specimen stage with a precision manipulator, and a two-dimensional X-ray charge-coupled device (CCD) camera. The CCD camera was set with an isotropic voxel resolution of 4.98 μm for all the samples. The bone samples were wrapped in Parafilm^®^ (American National Can™, Chicago IL, USA), and placed on a brass stub with Playdough. Scans were obtained at 70 kV and 129 μA using a 0.5 mm thick aluminum filter to optimize the contrast, a 360° rotation, three-frames averaging, a rotation step of 0.4°, and an exposure time of 560 ms. After reconstruction (NRecon^®^), the imaging analysis (CTan) of bone formation was done for both groups. We defined an 80 µm ring around implants (as the bone-implant interface) and a 1.6 mm circle (equal to the defect size) for the sham group. The 3D images were generated in CTVOx (Bruker micro-CT, Kontich, Belgium). Regarding the Mg group, we chose the 80 µm ring as the interface as this size would be enough to study the bone metabolism. In addition, when we chose bigger regions, we could reach the cortical bone in some animals (because of the anatomical differences between different animals), which could provide us some false positive results by analyzing the old bone tissue in the cortical area.

### Histological, enzyme histochemical, and immunohistochemical analyses

After µCT imaging, we embedded the fixed samples in Technovit^®^ 9100 new according to the manufacturer’s protocol (Heraeus Kulzer, Hanau, Germany).^59^ The samples were sectioned in 5 µm thickness onto Kawamoto’s film (SECTION-LAB Co. Ltd., Hiroshima, Japan) using a motorized rotary microtome (Thermo/Microm HM 355 S, Thermo Scientific GmbH, Karlsruhe, Germany). Movat Pentachrome and Von Kossa/Van Gieson stains were used to evaluate the bone mineralization/non-mineralization balance over time.

Movat Pentachrome stain was used to image different constituents of the connective tissue. The stain colors the tissues so that mineralized bone appears bright yellow, mineralized cartilage appears as blue-green, and non-mineralized bone, elastic fibers, and muscles appear bright red.^60^ Von Kossa/Van Gieson staining was used to distinguish the mineralized bone matrix from the newly formed bone matrix. The stain indicates mineralized bone matrix in black and newly formed bone matrix in pink to red color.^61^

To study the osteoblast and osteoclast balance, we used alkaline phosphatase (ALP) and tartrate resistant acid phosphatase (TRAP) enzyme histochemistry as known biological markers for osteoblast and osteoclast activities, respectively. Briefly, sections were deplastified, for TRAP treated with Sodium Acetate buffer and incubated in Napthol-AS-TR phosphate (N6125-1G, Sigma, Germany) and Sodium Tartrate (Merck, Germany) at 37°C for 60 min. For ALP stained sections, samples were treated with Tris and then incubated in BCIP/NBT phosphate substrate at 37°C for 60 min.^62,63^

Collagen fibers properties (such as width, length, straightness, and angle) were evaluated using Sirius Red. The imaging was done using a polarized filter.

Immunohistochemistry was performed using primary antibodies (Abcam Company, Cambridge, UK). The following antibodies were used: rabbit monoclonal [EPR5368] to alpha smooth muscle Actin (α-SMA), rabbit monoclonal [EPR24039-262] to factor VIII, rabbit polyclonal to CD68 (ab125212), rabbit polyclonal to CD80 (ab64116), rabbit polyclonal to Mannose Receptor, also known as CD206, (ab64693), rabbit monoclonal [EPR14335-78] to SRY-Box transcription factor 9 (Sox 9), rabbit monoclonal [EPR14334] to runt-related transcription factor 2 (Runx 2). Readers can find the details of immunohistochemistry protocol and materials elsewhere.^63,64^

To study the blood vessel formation and neo-vascularization, α-SMA and factor VIII primary antibodies were diluted in DAKO-Diluent (S 0809), 1:2000 and 1:1000, respectively. Regarding the macrophage polarization study, CD68, 80, and 206 were diluted in DAKO-Diluent, 1:200, 1:300, and 1:500, respectively. For type 1 macrophages, we used CD68 and CD80 as the marker set as well as CD68 and CD206 for type 2 macrophages.^63^ Furthermore, Sox 9 and Runx 2 were diluted in DAKO-Diluent (1:500) to study the bone metabolism.

We chose Movat Pentachrome and α-SMA stained sections to study the general tissue formation descriptively. In the Movat Pentachrome stained sections, we studied the tissue homogeneity and integrity as well as defect closure in both groups using a 3-point scale system (poor, fair, good for 1–3, respectively). Additionally, α-SMA stained sections were used to study the blood vessel phenotype and regularity over time using a 3-point scale system. We defined the round shape vessels as regular type 1, small to moderate oval shape ones as regular type 2 and big vessels in oval or other undefined shapes as irregular type 3 vessels.

### Quantitative histomorphometrical analysis

Imaging was done using a Leica microscopy system (Leica DM5500 photomicroscope equipped with a DFC7000 camera and operated by LASX software version 3.0, Leica Microsystem Ltd, Wetzlar, Germany). All stained sections (except from TRAP and Sirius Red) were imaged at 20× (3.09 pixel/μm) magnification. TRAP and Sirius Red stained sections were imaged at 40× (6.17 pixel/μm) magnification.

The histomorphometry measurements of Sirius Red stained sections were done using CT-FIRE (Curvelet Transform and Fiber Extraction Algorithm) software developed in Matlab (The Mathworks Inc.). This software automatically allows extracting collagen fibers in an image and quantifies the fiber properties with descriptive statistics (such as fiber angle, length, straightness, and width).^65^

Fiji ImageJ was used for histomorphometry measurements of other stained sections. Fiji ImageJ (version 1.51r; NIH, Maryland, USA) was used as a platform to run the program. The Trainable Weka Segmentation (TWS) was used as the base to create an optimized script to analyze tissue formation parameters such as mineralization, new bone and cartilage formation, vascularization and macrophage polarization. The histomorphometry measurements were done following Malhan et al.^66^ paper.

### Small angle X-ray scattering (SAXS) analysis

Technovit embedded bone sections with 70 µm thickness were provided to study the collagen/hydroxyapatite (HAp) orientation and the size of hydroxyapatite plates using SAXS analysis. Sections were measured at a SAXS lab source, Nanostar, Bruker, Germany. The used wavelength was 1.54 Å, and the sample to detector distance was 1.083 m. The calibration of the instrument was done using silver behenate. For azimuthal integration pyFAI was used. Sections were raster-scanned with a beam of 60 µm diameter. The step size in the 2D scan was adjusted accordingly to 60 µm. The scanned regions were chosen based on microscope images, and the region size was also adapted to the section based on the histological and µCT images.

The analysis of HAp orientation and the size of hydroxyapatite plates was done using a Matlab script developed in-house. The orientation of the platelets was evaluated based on the anisotropic scattering signal. To determine the size and degree of orientation, the stack and card model developed by Gourrier et al.^67^ was used.

In the scattering data, no direct signal from collagen could be seen. However, anisotropic scattering originating from HAp could be identified. As HAp normally aligned with the collagen matrix in which they are mineralized, it is a reasonable assumption that the collagen matrix orientation can be deduced from the HAp orientation.

### Statistical analysis

A Kolmogorov–Smirnov test was done to check the parametric or nonparametric distributions of the dataset. Then, a normality test was done (Holm-Sidak method). When the data were distributed normally, the data were presented as arithmetic mean values with standard deviation; and as median values with interquartile range when the data were not normally distributed. One-way ANOVA on ranks was performed when the normality test failed, using the Kruskal–Wallis test for post hoc comparison. Otherwise, regular ANOVA was performed with a Tukey test for post hoc comparison. All analysis were performed in GraphPad Prism 8 (GraphPad Software Company, San Diego, California, USA). Significant and highly significant differences were presented as *p < 0.05 and **p < 0.01, respectively.

## Results

### Macrophage polarization

The bone healing process has three main phases including inflammatory, reparative, and remodeling phases, which partially overlap with each other. Bone injuries disrupt bone matrix, blood vessels, and the surrounding soft tissues, which consequences in bleeding into the defect gap. Bleeding forms the initial hematoma, which causes the hypoxic state around the defect gap.^68–70^ Hematoma is followed and accompanied by inflammation, which is started by the expression of various cytokines, growth factors, and extracellular matrix (ECM) proteins. The expression of these inflammatory markers stimulates, recruits, and supports the proliferation of essential cells for bone formation.^68–70^ For instance, Interleukin-1 (IL-1) and IL-6, which are secreted by macrophages and T cells, respectively, both stimulate proliferation and differentiation of MSCs into chondrocytes and osteoblasts after injuries.^71^ Hence, we first studied the vascularization and macrophage polarization after implantation.

**Figure 1.**
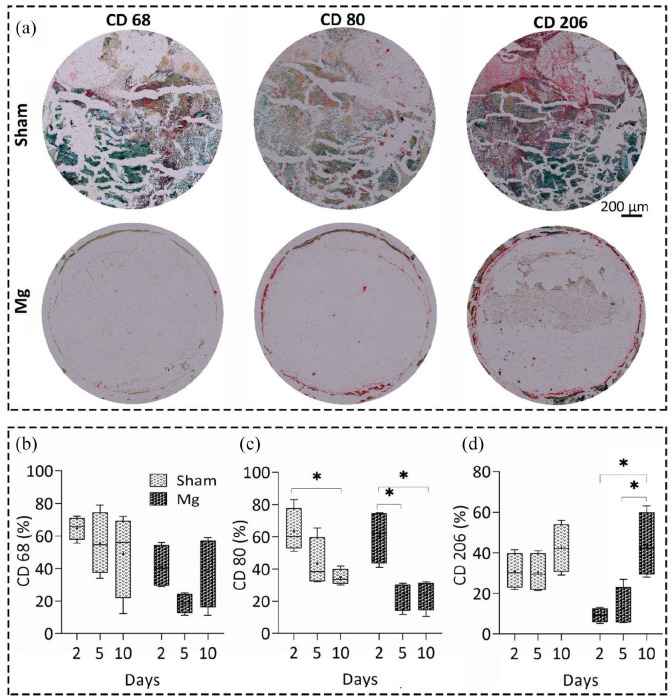
Immunohistochemical analysis of macrophage polarization over time. (a) Representative images of antibody staining against CD68, 80, and 206 macrophage markers (red color) 10 days after implantation in the Mg-based alloy and sham groups, scale bar = 200 µm. CD68, 80 were used as the markers of macrophage type 1, while CD80 and CD206 were the markers for macrophage type 2. Quantitative histomorphometrical data of macrophage polarization using the percentage of antibodies against CD68 (b), CD80 (c), and CD206 (d) markers over time. The percentage of CD80 decreased significantly after 10 days in both groups (*p* < 0.05). However, the percentage of CD206 marker significantly increased only in Mg group after 10 days, compared with day 2 and 5 (*p* < 0.05). After 5 and 10 days, macrophage phenotype changed in both groups from predominantly macrophages type 1–2 with significant changes for Mg group (b–d). We observed a significantly higher number of CD206 positive macrophages (type 2 macrophages) for Mg group compared with sham over time. Values represent the mean ± standard deviation. Significant differences were presented as *p < 0.05.

It should be noted that in this study, because of the different regions of interests of sham (defect site, 1.6 mm) and Mg groups (80 µm at the interface area), we did not directly compare the two groups to each other. We studied the tissue healing of both groups individually and compared the responses over time. We studied the macrophage polarization in both groups by focusing on the distribution of macrophage type 1–2 subsets over time (Figures 1(a) and S1-A). We used CD68 and CD80 subsets as the biological markers of type 1 macrophages, as well as CD68 and CD206 subsets for type 2 macrophages.^41^ During the first 5 and 10 days, macrophage phenotype changed in both groups from predominantly macrophages type 1–2 with significant changes for Mg group (Figure 1(a)–(d)). We observed a significantly higher number of CD206-positive macrophages (type 2 macrophages) for Mg group in comparison to sham over time. Type 2 macrophages were present 2 and 5 days after the surgery, and they dominated at the interface 10 days after surgery, while the percentage of type 1 macrophages was decreased significantly. Although the same pattern was seen in the sham group, the changes in type 2 macrophages were not significant indicating improved immunomodulatory effects of Mg alloy toward tissue healing.

### Vascularization

**Figure 2.**
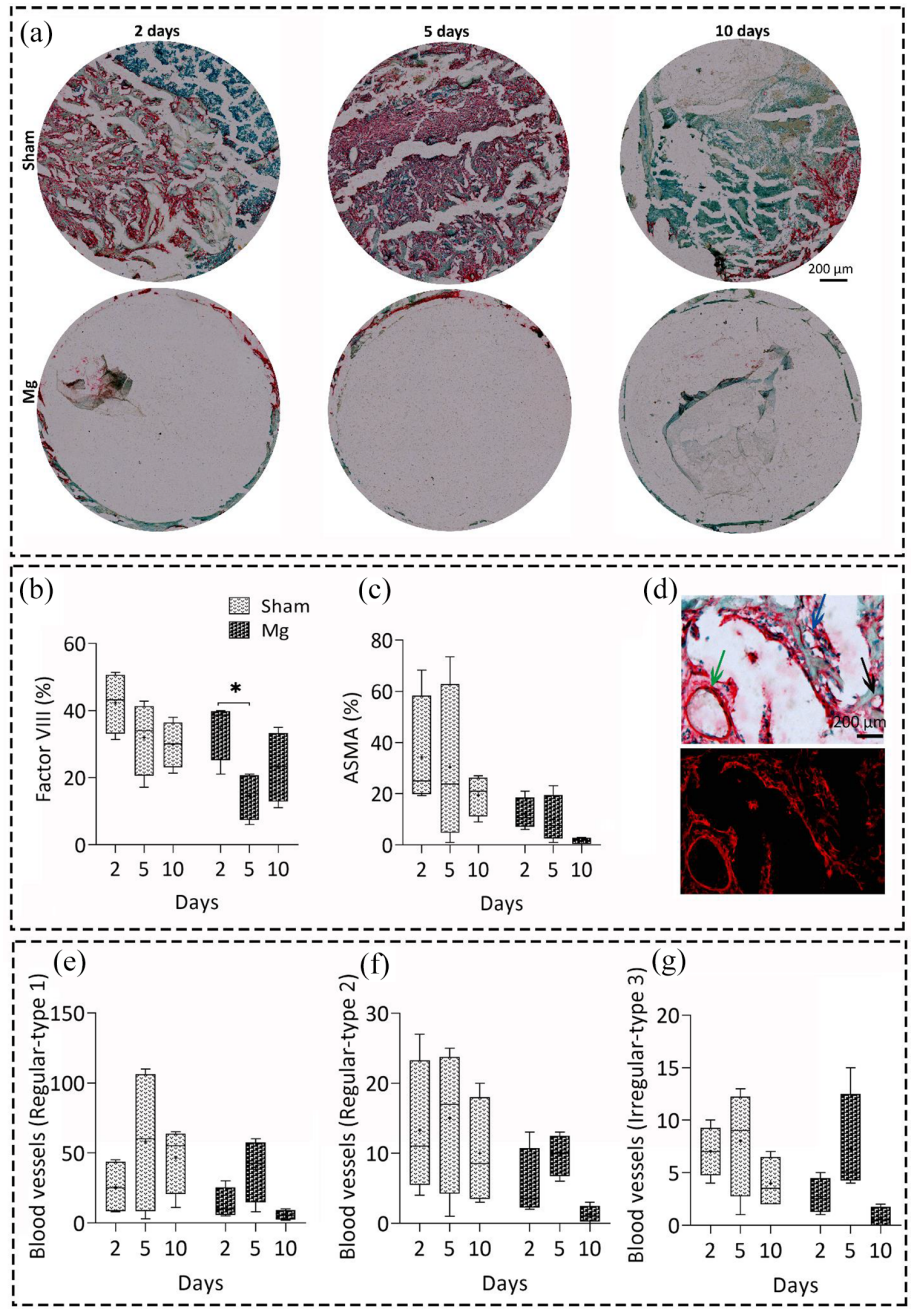
Immunohistochemical evaluation of early blood vessel formation and neo-vascularization of Mg-based alloy and sham groups over time. (a) Representative images of α-SMA antibody staining (red color) for early blood vessel formation 2, 5, and 10 days after implantation in both groups. Quantitative histomorphometrical data of neo-vascularization using Factor VIII antibody (b), and blood vessel formation using alpha smooth muscle Actin (α-SMA) antibody percentage over time, scale bar = 200 µm (c). An insignificant decrease in the percentage of positive blood vessels was observed for both factor VIII and α-SMA positive blood vessels over time in sham group. The percentage of factor VIII positive blood vessels decreased significantly for Mg group at day 5 compared to day 2 (*p* < 0.05). (d) Representative bright field and fluorescent images of α-SMA antibody staining used for evaluating the blood vessel regularity over time using a 3-point scale system, scale bar = 200 µm. The round shape vessels were categorized as regular type 1 vessels, black arrow (d, e), small to moderate oval shape ones as regular type 2, blue arrow, (d, f) and big vessels in oval or other undefined shapes as irregular type 3 vessels, green arrow (e, g). Values represent the mean ± standard deviation. Significant differences were presented as *p < 0.05.

We considered factor VIII and α-SMA positive blood vessel formation to study the neo-vascularization and blood vessel formation in both groups over time (Figure 2). The bright field and fluorescent representative mages of α-SMA positive blood vessels in both groups are shown in Figures 2(a) and S1-B, respectively. Regarding the sham group, the number of positive blood vessels decreased for both factor VIII and α-SMA positive blood vessels over time; however, the changes were not significant (Figure 2(b) and (c)). However, the regularity of α-SMA positive blood vessels increased over time, suggesting a normal tissue healing (Figure 2(d)–(f)). Although we found no significant differences for the sham group over time, the largest data variance was seen on day 5. The percentage of factor VIII positive blood vessels (for neo-vascularization) decreased significantly for Mg group at day 5 (*p* < 0.05) and increased at day 10. However, the α-SMA positive blood vessels had an opposite pattern at day 5, with an increase in the number of positive vessels, regardless of their regularity type (Figure 2(b)–(f)).

### Bone mineralization

**Figure 3.**
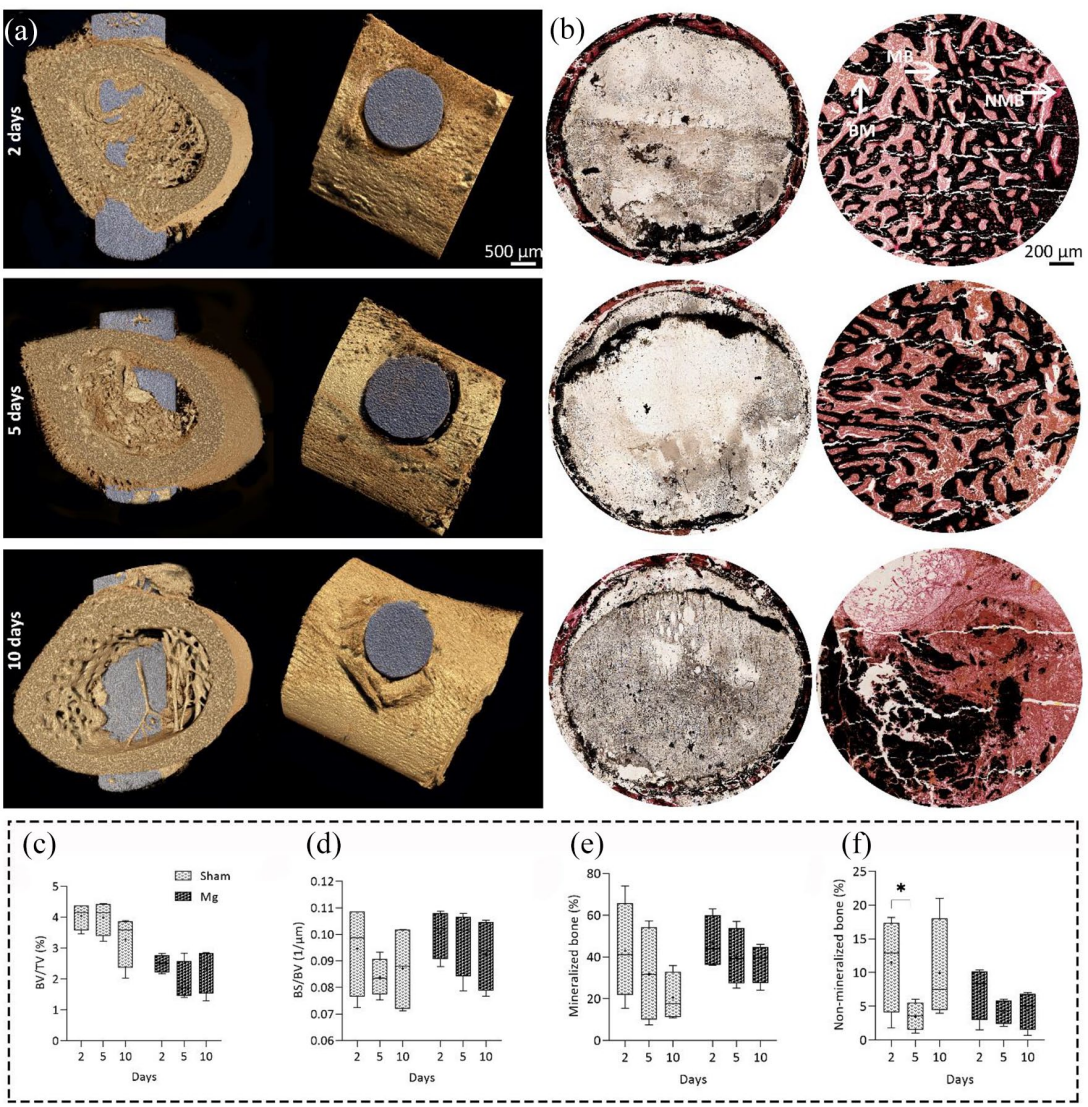
Analyzing the bone mineralization in Mg-based alloy and sham groups over time. (a) Representative images of 3D µCT analysis of bone mineralization in Mg group over time, in the transverse and coronal planes of µCT (left and right panels, respectively), scale bar = 500 µm. (b) Representative images of 2D evaluation of mineralized (MB, black color) versus non-mineralized bone matrix percentage (NMB, pink to red color) in both groups over time, scale bar = 200 µm. The brown color represents bone marrow (BM). Quantitative data of bone volume to tissue volume percentage (BV/TV) (c) and bone surface to bone volume (BS/BV) (d) using 3D µCT analysis. The BV/TV and BS/BV did not change significantly over time for both groups. Quantitative histomorphometrical data of mineralization (e) and new bone formation (f) using Von Kossa/Van Gieson staining. Changes in mineralization and non-mineralization using Von Kossa/Van Gieson staining were also insignificant in both groups. However, changes in the non-mineralization had more fluctuations in both groups, by decreasing and increasing after 5 and 10 days, subsequently. Values represent the mean ± standard deviation.

We studied the mineralized versus non-mineralized bone formation over time, using both 3D µCT and 2D histology and compared data afterward (Figures 3(a), (b), and S2). We observed no significant differences over time for both groups regarding the percentage of bone volume to tissue volume (BV/TV) and bone surface to bone volume (BS/BV). In terms of mineralization, the 2D histology data from Von Kossa/Van Gieson staining confirmed the µCT data indicating no significant differences over time for both groups. The non-mineralization (newly bone matrix formation) decreased after 5 days and then increased after 10 days in both groups; however, the changes were not statistically significant. The sudden changes in the bone matrix formation of both groups at day 5 were in accordance with the sudden changes of vascularization and macrophage polarization data.

**Figure 4.**
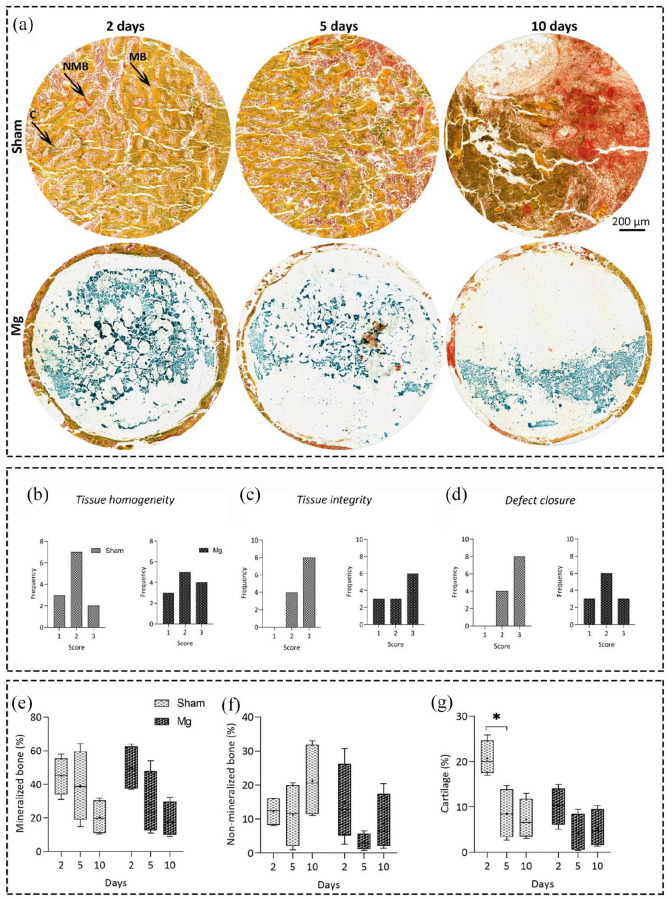
Analyzing the bone mineralization and cartilage formation in Mg-based alloy and sham groups over time. (a) Representative images of Movat Pentachrome histology staining. Mineralized (MB) and non-mineralized bone (NMB) as well as cartilage formation (c) were evaluated in both groups over time, scale bar = 200 µm. Descriptive analysis of tissue homogeneity (b) and integrity (c) as well as defect closure (d) in both groups using a 3-point scale system (good, fair, poor for 1–3, respectively). Quantitative histomorphometrical data of mineralization (e), non-mineralization (f) and cartilage distribution percentage (g) using Movat Pentachrome histology staining. There was no significant differences between groups regarding their tissue homogeneity, integrity, and defect closure. Although the percentage of mineralized and non-mineralized bone tissue did not significantly change over time for both groups, cartilage formation significantly decreased after 5 days for sham group (*p* < 0.05). Values represent the mean ± standard deviation. Significant differences were presented as *p < 0.05.

We further studied the bone formation changes over time using Movat Pentachrome staining and analyzed the changes both quantitatively and descriptively (Figure 4). The descriptive data showed that in the majority of sham samples, the tissue homogeneity was in the bad to fair categories (1 and 2), regardless of time point, while in the fair to good categories (2 and 3) for Mg-based alloys. The tissue integrity and defect closure of all sham samples and the majority of Mg-based alloys were in the fair to good categories (2 and 3). These data supported the ZX00 alloy stimulatory potential toward normal bone healing.

Regarding bone mineralization, the quantitative data from this staining confirmed the previous results obtained by µCT and Von Kossa/Van Gieson staining. In addition, cartilage distribution decreased significantly for sham group after 5 days with *p* value of 0.0350. The cartilage formation is a key part of the bone healing reparative phase. This phase is mainly addressed by the development of new blood vessels and cartilage formation. The neighboring soft tissue stimulates vascular ingrowth firstly to the periosteal area and then to the endosteal layers of tissue (Figure 4).^72,73^ In the normal conditions, the cortical blood supply is mainly from endosteal bone and branches out in a radial manner from the center of the medullary canal. However, during the reparative phase, most of the cortex blood supply comes from outside the tissue than inside of it. Inflammatory mediators in the fracture hematoma activate chondrocytes to form the fracture callus. Hematoma is ultimately substituted by the ingrowth of fibrovascular tissue. This developing construct supports the stabilization of bone ends. At this stage, proteins secreted by osteoblasts and chondroblasts consolidate into a new bone substance named as a *soft callus*, which eventually strengthens into a *hard callus* as the bone forms its final texture. The two types of bone formation known as intramembranous and endochondral bone formation can occur during the first 10 days of injuries.^72,73^ The endochondral bone formation takes place in the absence of rigid fixation.^72,73^ Differentiation of progenitor cells into chondrocytes following by the secretion of biological factors leads to producing a cartilaginous matrix, including collagen II. This *soft callus* spans the fracture gap.^68,74,75^ Later chondrocytes undergo hypertrophy, and chondrocyte-mediated mineralization, in a process similar to the one that occurs during the development of growth plate.^76^ When vasculature starts to invade, the hypertrophic chondrocytes are removed and woven bone formation occurs after the recruitment of osteo-progenitor cells.^77^

### Bone metabolism and Mg retention

**Figure 5.**
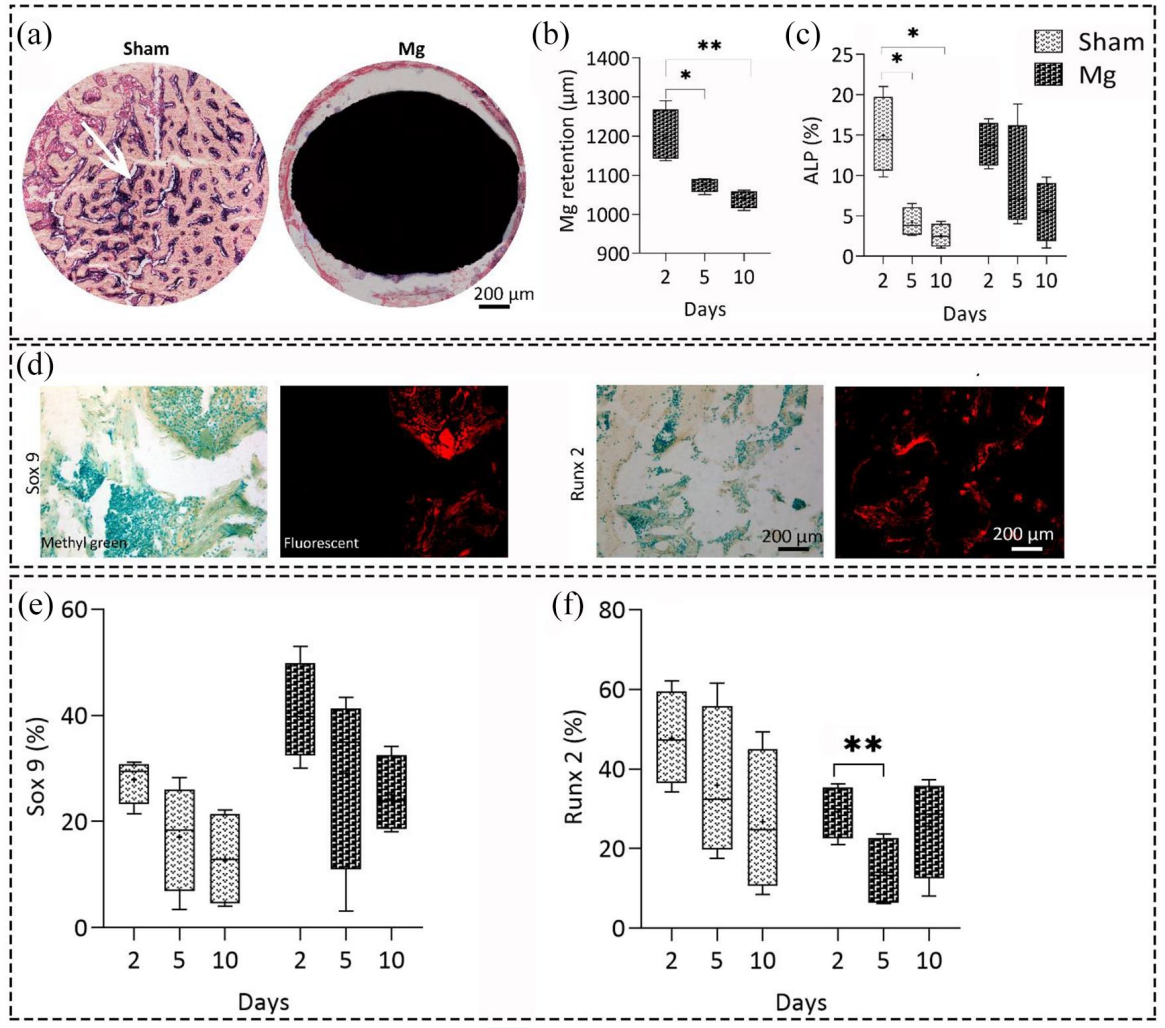
Enzyme histochemical and immunohistochemical analyses of bone metabolism activities in Mg-based alloy and sham groups over time. (a) Representative images of osteoblast activity (purple color) using alkaline phosphatase (ALP) enzyme histochemistry in Mg-based alloy and sham groups 10 days after implantation. (b, c) Quantitative data of the Mg-based alloy retention and osteoblast activity over time based on ALP stained sections, respectively. (b) Mg had a significant degradation after 5 and 10 days compared to day 2 with *p* values of *p* < 0.05 and *p* < 0.01, subsequently. (c) The ALP activity decreased significantly after 5 and 10 days for sham group (*p* < 0.05) indicating less osteoblast activity; however, changes were not significant for Mg group. (d) Representative images of immunohistochemistry staining against SRY-Box Transcription Factor 9 (Sox 9) and runt-related transcription factor 2 (Runx 2) biological markers, scale bar = 200 µm. (e, f) Quantitative histomorphometrical data of Sox 9 and Runx 2 biological markers percentage. (e) An insignificant decrease in the percentage of Sox 9 was observed in both groups over time. (f) Although the percentage of Runx 2 decreased significantly after 5 days (*p* < 0.01) in Mg group, it started to increase after 10 days. Values represent the mean ± standard deviation. Significant and highly significant differences were presented as *p < 0.05 and **p < 0.01, respectively.

We further studied the bone healing process in both groups by focusing on the balanced osteoblast/osteoclast activities as well as the balanced anabolic and catabolic responses after implantation (Figure 5). During the last phase of bone healing process, known as remodeling, the newly woven bone is converted into the lamellar bone. First, osteoclasts begin absorbing a cavity known as the cutting cone. Osteoblasts migrate to this cone and form a bone matrix layer in opposition to the existing surface, which ultimately leads to regenerate the original structure and biomechanical competence of the injured sites.^78,79^ Therefore, here we focused on evaluating the osteoblast/osteoclast activities. We observed a significant decrease in the osteoblast activity using ALP enzyme histochemistry after 5 and 10 days in sham group compared to day 2, with *p* values of 0.0189 and 0.0271, respectively. In the Mg group, there were no significant differences between the ALP activity of defined time points (with *p* values of 0.6416, 0.0908, and 0.4355 for 2 vs 5, 2 vs 10, and 2 vs 10 days, respectively) (Figure 5(a) and (c)).

We also used TRAP enzyme histochemistry to study the osteoclast activity. Although some research groups observed the osteoclast activity after 1 week in vitro and/or in vivo using TRAP staining,^80,81^ we did not see any osteoclasts in the defined region of interests of both groups (data not shown). The Mg retention over time was done by comparing the Mg diameter before (1.6 mm) and after implantation (Figure 5(b)), we utilized the same histological sections that were used for the ALP staining. The integrity of Mg-based alloy structure was favorable in those sections than the TRAP stained ones. The data showed a significant Mg retention 5 and 10 days after the implantation compared to day 2. However, the differences between 5 and 10 days after implantation were insignificant.

To study the bone metabolism activities after implantation in more detail, we evaluated the percentage of Sox 9 and Runx 2 as the key players in determining the chondrocyte and osteoblast cell fate, respectively (Figure 5(d)).^82^ Regarding Sox 9, the changes were not statistically significant and only a slight decrease was seen over time for both groups (Figure 5(e)). However, the total percentage of chondrogenesis in Mg group was higher than that of sham group. The percentage of Runx 2 biological marker for osteoblast activity did not significantly change over time for sham group (Figure 5(f)); however, it significantly decreased for Mg group at day 5 (*p* value = 0.0094). The Runx 2 percentage increased slightly at day 10, which could be a sign for initiating the bone formation mechanisms after 10 days compared to day 5.

### Collagen/hydroxyapatite (HAp) orientation

**Figure 6.**
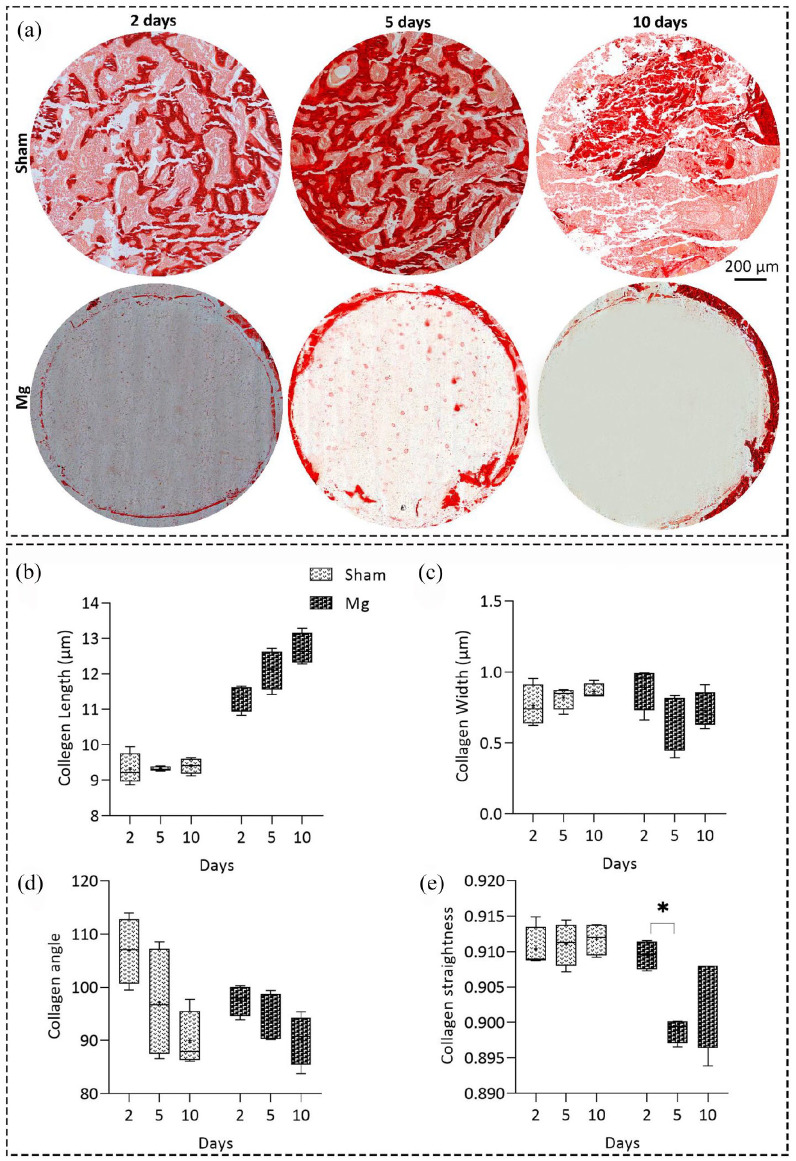
Studying collagen fiber properties in the Mg-based alloy and sham groups over time. (a) Representative images of Sirius red staining 2, 5, and 10 days after implantation in both groups, scale bar = 200 µm. Quantitative histomorphometrical data of collagen fiber length (b), width (c), angle (d), and straightness (e) using Sirius red staining. Although the collagen fiber length, width, and straightness remained almost unchanged over time for the sham group, we observed increasing the fiber length as well as decreasing the fiber width, angle, and straightness toward 90° orientation. A significant decrease in the collagen straightness for Mg group was observed after 5 days (*p* < 0.05). Values represent the mean ± standard deviation. Significant differences were presented as *p < 0.05.

The collagen architecture is a key player in determining the function and mechanical behavior of bone tissue. To engineer a functional and load-bearing bone tissue that meets the body’s mechanical demands, we require a detailed understanding of the collagen orientation and properties after implantation compared to the normal healing conditions after a fracture. Using Sirius Red staining, we also studied the collagen fiber properties in both groups over time (Figure 6(a)). The collagen fiber length, width, and straightness (Figure 6(b), (c), and (e)) remained approximately unchanged over time for the sham group. However, regarding the Mg-based alloy group, we observed increasing the fiber length as well as decreasing the fiber width, angle, and straightness toward 90° orientation (Figure 6(b)–(e)).

**Figure 7.**
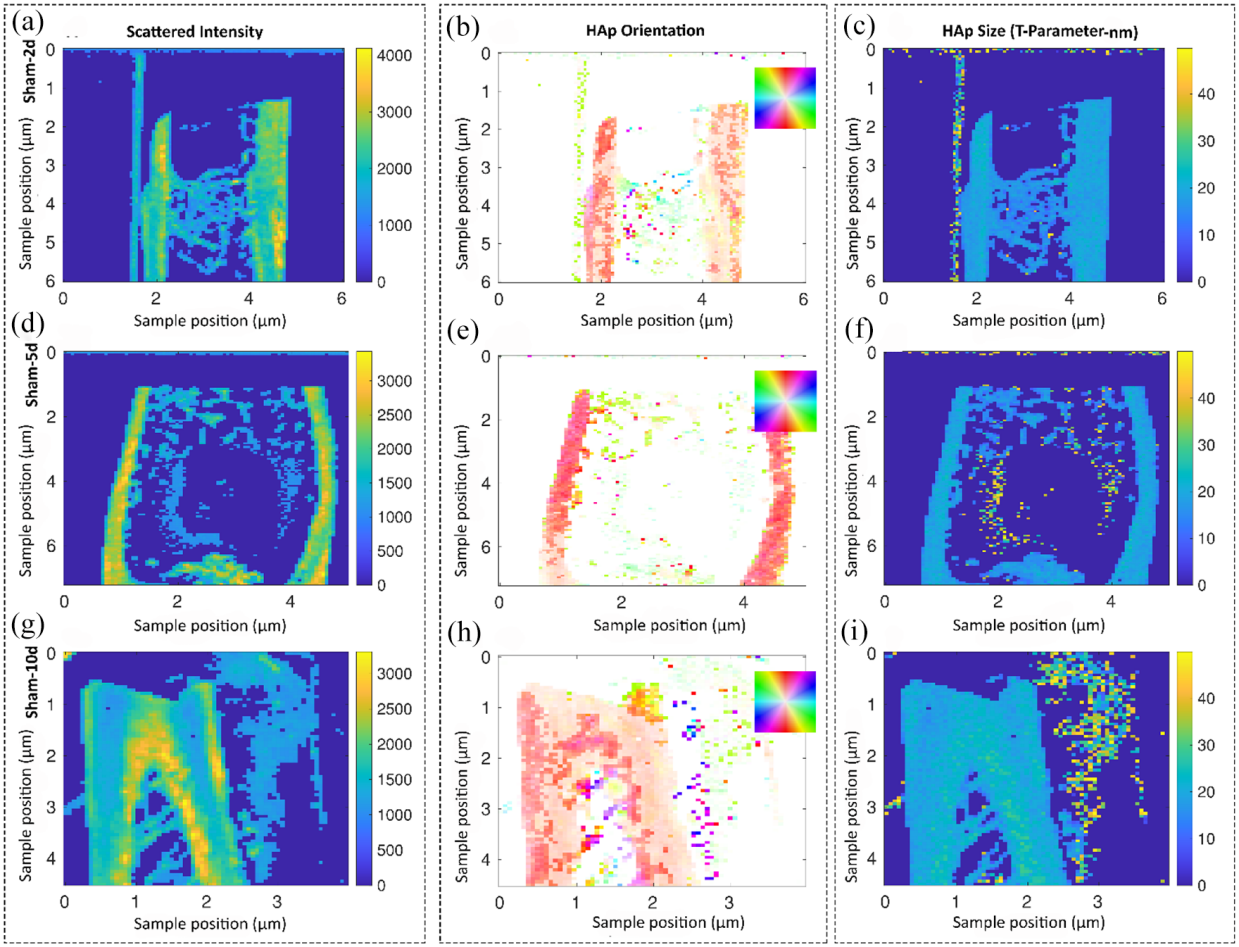
Studying the collagen/hydroxyapatite (HAp) orientation and the size of hydroxyapatite plates in the sham group over time using small angle X-ray scattering (SAXS) analysis. Samples are scanned in a region of approximately 5 mm by 5 mm and each pixel corresponds to a probed region of 60 µm in radius. The shown information are a result of evaluating the scattering data and, therefore, test specific features at other length scales. Representative images of descriptively analyzing the scattered intensity (a, d, g) as well as the HAp orientation in degree (b, e, h) and size of the platelets in nm (c, f, i). No residuals of the defect were detected after day 10 for sham group (g, h). The HAp size was homogenous along the bone and around 2 nm for all time points (c, f, i). The color code in the HAp orientation analysis (b, e, h) shows the orientation degree, which corresponds to the inset, for example, red is along the *y*-axis of the image.

**Figure 8.**
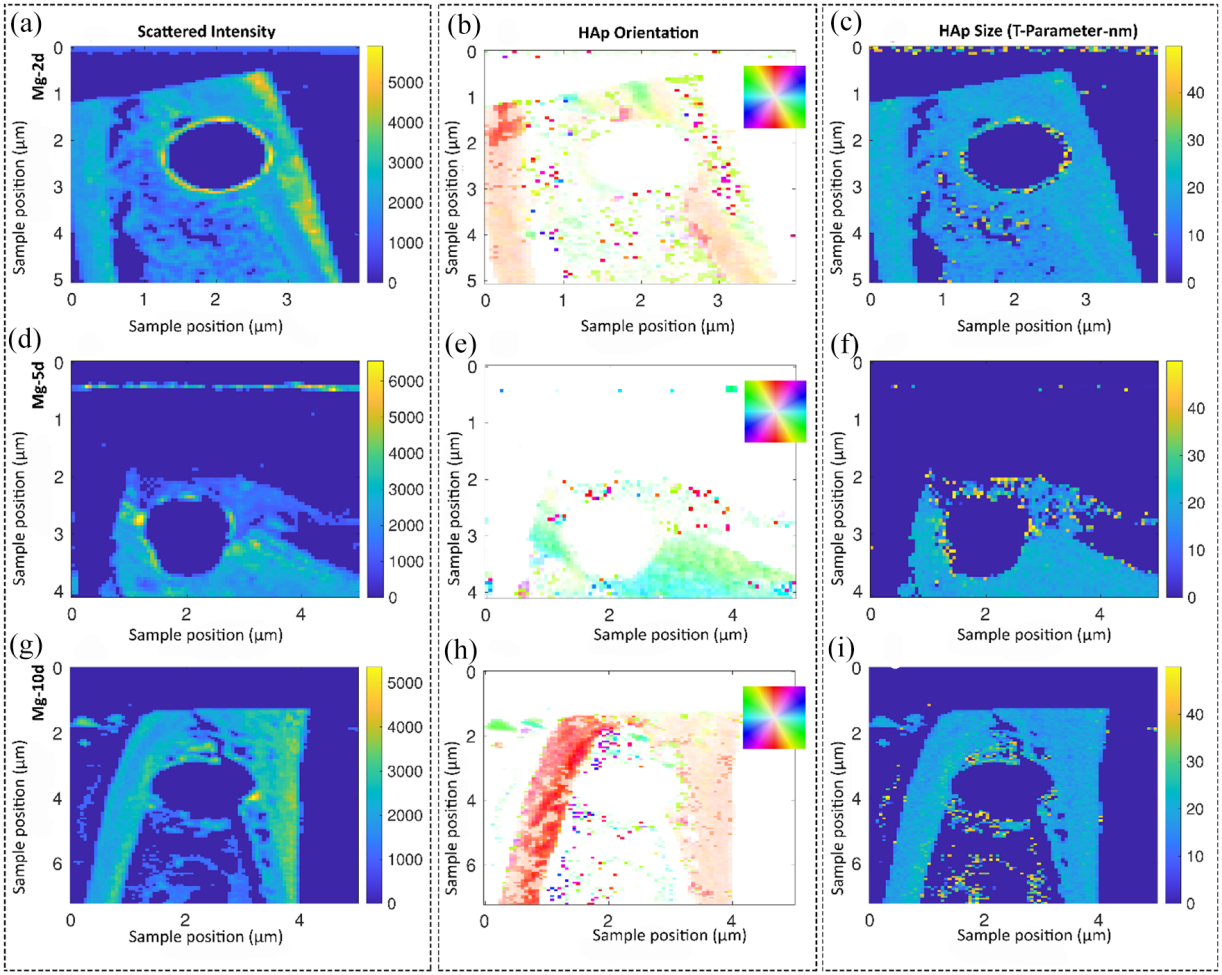
Studying the collagen/hydroxyapatite (HAp) orientation and the size of hydroxyapatite platelets in the Mg-based alloy group over time using small angle X-ray scattering (SAXS) analysis. Samples are scanned in a region of approximately 5 mm by 5 mm and each pixel corresponds to a probed region of 60 µm in radius. The shown information are a result of evaluating the scattering data and, therefore, test specific features at other length scales. Representative images of descriptively analyzing the scattered intensity (a, d, g) as well as the HAp orientation in degree (b, e, h) and size of the platelets in nm (c, f, i). Only a minor degree of HAp orientation was observed at the Mg interface after 5 days (d–f). However, the HAp platelets size increased at the interface. The bone orientation at day 5 was in the horizontal direction; however bone was always orientated vertically at other time points. The color code in the HAp orientation analysis (a, d, g) shows the orientation degree, which corresponds to the inset, for example, red is along the *y*-axis of the image.

Although histological and immunohistochemical analyses are considered the gold standard experiments to study the biomaterial-tissue interface, it would be beneficial to use other imaging systems to study their potential in evaluating the interface. Therefore, besides histological, enzyme histochemical, immunohistochemical and histomorphometrical analyses, we evaluated the new bone formation at the interface using 3D micro computed tomography (µCT) and small-angle X-ray scattering (SAXS) analyses. Using SAXS, we analyzed the collagen/hydroxyapatite (HAp) orientation and the size of hydroxyapatite plates (T-parameter) to study the effects of Mg-base alloy on the quality of formed bone at early time points compared to the sham group (Figures 7 and 8). Figure 7 shows the scattered intensity as well as HAp orientation and size of the sham group over time. The cortical region of bone had an orientation along the defect axis 2 and 5 days after surgery (Figure 7(a) and (f)). In the medulla (where the defect site was located), a mineralized region was presented 2 days after surgery; however, it was much larger than the original defect size (Figure 7(a) and (b)). We could also observe the mineralized regions in the cortical bone region 5 days after surgery (Figure 7(d) and (e)). Although the HAp/collagen matrix was in the orientation along the bone axis, we could not observe any residuals of the defect at day 10 (Figure 7(g) and (h)). The size of HAp was roughly 2 nm for all time points (Figure 7(c), (f), and (i)).

Figure 8 shows the scattered intensity as well as HAp orientation and size of the Mg-based alloy group over time. The implant location was visible in all samples. Figure 8(a) shows a high-intensity scattering ring around the implant, which is mostly originating from the implant located in the sections. The most orientated regions were located at the cortical bone of sample in the direction of the bone axis. Although we observed less orientation of the HAp crystals around implants, a highly mineralized region could be seen as a scattering signal at the interface (Figure 8(a)–(c)). We observed only a minor degree of HAp orientation at the interface on day 5 (Figure 8(d)–(f)). However, the HAp platelets size increased at the interface. The bone orientation at day 5 was in the horizontal direction compared to the other time points, where the bone was always orientated vertically. This could be a reason for the previously observed histological changes after 5 days in Mg group. The scattering distribution in Figure 8(g) directly shows the implant position 10 days after implantation. The scattering data showed a mineralized bone layer of roughly 120 µm at the interface. Even though we observed a strong scattering signal at the interface, a minor degree of orientation was seen. We detected the strongest orientation of HAp and collagen in the cortical bone showing an orientation parallel to the bone axis on day 10.

Overall, the size of HAp crystals along the cortical bone was roughly 2 nm. We observed an increased HAp thickness (3.5–4 nm) at the bone-implant interface, indicating better bone remodeling. These results were in accordance with the data obtained from Sirius Red staining indicating better collagen fiber orientation and bone quality in Mg group compared to sham.

## Discussion

### Mg degradation and gas formation

Owing to their biodegradability, Mg-based implants have gained substantial attention in the medicine world as a replacement for permanent bio-inert metallic implants.^46,52,83^ Mg degrades through a corrosion process initiating from its standard electrode potential of −2.372 V versus the normal hydrogen electrode. It corrodes in aqueous solutions by forming magnesium hydroxide (Mg(OH)_2_) and hydrogen gas (H_2_).^84^ The corrosion products (mainly Mg^2+^ ions) interact with chloride ions in the body fluid or are digested by macrophages.^49^ However, during the early stages of Mg implantation, it corrodes quicker, causing an implant mechanical instability. This occurs owing to the lack of MgO + Mg(OH)_2_ protective layer on the fresh surface.^85^ Recently, Grün et al.^50^ developed the ZX00 alloy, as a new Mg-based alloy to avoid the rapid corrosion of Mg.^50^ They studied the long-term bone tissue responses to the alloy in both growing-rat and sheep models to investigate its potential for improving pediatric bone healing. Their results indicated a slow and homogeneous alloy degradation with an average degradation rate of 0.08 and 0.045 mm/year in rat and sheep animal models, respectively.^50^ In this study, we calculated the Mg degradation rate by comparing the diameter of the XZ00 alloys in the histological sections with the initial diameter of alloys before implantation. We observed a significantly fast Mg surface retention by measuring the diameter of pins after implantation within the first 5 days of our study. The same growing-rat animal model indicated the possible influence of biochemical interactions between the Zn, Ca, and Mg ions with the bodily fluids. However, the degradation rate remained stable between 5 and 10 days of implantation.

Previously, Walker et al.^86^ Studied the degradation rate of pure Mg and five alloys (AZ31, Mg-0.8Ca, Mg-1Zn, Mg-1Mn, Mg-1.34Ca-3Zn) in vitro and in vivo in the subcutaneous environment of rats after 7, 14, and 21 days of implantation.^86^ They observed that the degradation rate of pure Mg, AZ31, and Mg-1Zn remained stable or slightly decreased between 7 and 14 days; however, Mg-0.8Ca, Mg-1Mn, and Mg-1.34Ca-3Zn degraded faster within the same time point.^86^ In addition, Fischerauer et al.^87^ studied the effects of micro-arc oxidation (MAO) surface treatment on the degradation rates of Mg-5Zn-0.3Ca alloy (ZX50) and in a rat animal model.^87^ Although using in vivo µCT, the corrosion layer could be observed on the ZX50 surface, the MAO-coated alloys are stable and do not show any sign of degradation after 7 days of implantation.^87^ In another study, Nidadavolu et al.^88^ reported that although the degradation rate of Mg-0.3Ca is fast within the first 24 h of immersion in DMEM Glutamax +10% FBS (Fetal Bovine Serum) +1% Penicillin streptomycin cell culture solution, it reaches a stable rate of 0.51 µm/day after 5 days.^88^ Studying the degradation rate of Mg alloys is still a challenging issue as it could be different depending on the in vitro and in vivo experimental conditions, the time the alloy is kept in formalin after harvesting the tissue, the Mg alloy composition, concentration of each element, and the used technique for evaluation.^89^

Within the studied time points, we did not observe any sign of H_2_ gas formation around the implant. However, this could not certainly mean that this alloy does not form any H_2_ gas. Grün et al.^50^ studied the gas formation of this alloy between 2 and 24 weeks in the same animal model and reported a moderate gas evolution 4 and 6 months after implantation.^50^ The alloy degradation rate and gas evolution should be further studied by considering longer time points and using in vivo µCT imaging to obtain detailed information.

### Inflammatory responses and vascularization

Because macrophages and other inflammatory immune cells play key roles in determining the biomaterial’s success after implantation, biomaterials with immunomodulatory potential have gained much attention over the past few years.^90–92^ Qiao et al.^25^ Have more recently demonstrated that the influx of magnesium through the transient receptor potential cation channel member 7 (TRPM7) channel and the nuclear translocation of TRPM7-cleaved kinase fragments (M7CKs) lead to the polarization of macrophages into a pro-osteogenic subtype.^25^ This further stimulates the osteogenic differentiation of MSC, which is distinct from the classical macrophage type 1/2 phenotypes. Their data supported that the initial immune response to bone injuries, regulated by cell types in the monocyte-macrophage-preosteoclast lineage, could be controlled to enhance tissue regeneration through different signals such as magnesium ion signaling pathways. However, the osteo-promoting functions of magnesium only is affective during the early phase of osteogenesis as the continued stimulation of magnesium over activates the nuclear factor kappa light chain enhancer of activated B cells (NF-κB) signaling pathway and prevents mineralization of extracellular matrix.^25^ Our results supported their findings indicating that ZX00 alloy could potentially modulate the early inflammatory responses after implantation toward tissue healing. We found that the alloy could significantly regulate the polarization of macrophages toward higher expression of type 2 macrophages, 5 and 10 days after implantation, which could stimulate tissue healing and reduce the immune responses against the alloy degradation products. We also observed significantly improved immune system responses to ZX00 alloy compared to sham, which supported our null hypothesis. This could be because of the early surface degradation of alloy and consequently the release of ions (Mg, Zn, and Ca ions) from the alloy surface and their potential interactions with intracellular ionic channels.^7,53^ This was in agreement with results from Li et al.^21^ study on macrophage responses to the Mg-coated titanium implants compared to pure titanium implants.^21^ They observed a significantly higher number of type 2 macrophages in the Mg-coated group 4 and 7 days post implantation.^21^ Mg is a Ca antagonist and can interfere with the Ca signaling pathways.^23,93^ It can control inflammation through activating phagocytic cells and their effector functions, intercellular Ca channels, N-methyl-d-aspartate (NMDA) receptors, and nuclear factor-kappa B (NFκB).^94^

After the implantation of a biocompatible implant, native blood vessels disrupt causing interactions between blood and implant.^18^ After the macrophage polarization from type 1 to 2, macrophages locally release several growth factors (e.g. vascular endothelial growth factor) and induce fibroblast and endothelial cell migration as well as proliferation by sending biochemical signals. The migrated endothelial cells promote the formation of new blood vessels toward neo-tissue formation.^95^ Our results indicated a slight increase in the percentage of blood vessels after 5 days in the ZX00 alloy group. Because neo-vascularization is a key player in stimulating bone healing.^21^

### New bone formation

In this study, we focused on the early osteogenic responses to ZX00 alloy by studying the mineralized versus non-mineralized bone matrix formation, osteoblast and osteoclast activities as well as balanced anabolic and catabolic responses. Regarding mineralization, our µCT and histology results indicated no significant differences over time for both groups. The newly bone matrix formation obtained from Von Kossa/Van Gieson staining decreased at day 5 and then increased within the next 5 days; however, no significant differences were observed. We focused on the activities of ALP and TRAP enzymes to further investigate the early bone healing. The osteoblast activity decreased significantly over time for sham group. However, it did not change substantially over time for ZX00 alloy group. We investigated the osteoclast activity using TRAP enzyme histochemistry; however, we did not observe any osteoclast in the defined region of both groups (data not shown). Some research groups observed the osteoclast activity after 1 week in vitro and in vivo studies using TRAP staining^80,81^; however, we did not see any osteoclast in the region of interests of both groups (data not shown). This could be because of delays in the osteoclast migration from the cortical bone to the defect site.

Studying the balanced anabolic and catabolic responses to the ZX00 alloy implantation compared to sham group, could provide us more details about the alloy potential in stimulating early osteogenic formation.^82^ Hence, we studied Runx 2 and Sox 9 activities as two major transcription factors in directing chondrocyte and osteoblast cell fates, correspondingly.^82^ The expression of Sox 9 biological marker did not significantly change over time for both groups. Although Runx 2 activity did not change significantly over time for sham group, it significantly decreased for XZ00 alloy at day 5 and then slightly increased after 10 days, indicating the initiation of bone formation mechanisms after 10 days. This also suggests that longer time points should be considered in future studies for better evaluation of bone formation mechanisms.

### Bone quality

Studying the collagen fiber properties (such as length, width, and alignment) provides useful information about the effects of implant surface properties on the distribution of stress applied to bone and the quality of bone tissue matrix at the interface.^96^ We could obtain some information on collagen fiber properties using Sirius Red histology staining and SAXS analyses. Our histology results indicated improved collagen orientation and alignment over time for the Mg-based alloy (increased fiber length as well as decreased fiber width and angle), suggesting better bone remodeling in this group compared to sham. These results were in line with SAXS results at day 10, when we detected the strongest orientation of HAp and collagen fibers in the cortical bone, presenting an orientation parallel to the bone axis. The HAp thickness at the bone-implant interface increased to almost twice (3.5–4 nm) the size of those in the sham group (2 nm), indicating bone remodeling after Mg alloy implantation. This was in line with Grün et al.^50^ Study, where thicker cortical bone structures were detected at the interface, 6 weeks after ZX00 alloy implantation compared to the sham group.^50^

### Animal model and implant shape

ZX00 alloy is mainly designed for pediatric orthopedic trauma.^7,50^ Because the bone metabolism varies between adults and children, to achieve reliable results, we should test such degradable implants in the juvenile growing-animal models. The bone turnover rate of animal models is more close to children.^50^ Grün et al.^50^ developed a growing-rat and—sheep animal model for this purpose and concluded that the bone healing results in both animals are comparable to that of children.^50^ Hence, we used the same animal model to evaluate the early inflammatory responses to the XZ00 alloy developed by Grün et al.^50^

### Study limitations and future perspectives

In this study, we focused on the macrophage polarization and its potential effects on osteogenic responses to implants by considering three early time points (2, 5, and 10 days post implantation). Hence, we cannot make any statement regarding the long-term outcome of ZX00 implantation. Although some studies reported its bone remodeling potential after 1–6 months of implantation,^7,50^ more in vivo studies are essential to evaluate its effects after 1 year to complete degradation.

In the case of degradable Mg as a tissue-regenerated biomaterial, not only wound healing processes, but also several other physiological, mechanical, and biochemical pathways are involved, which could play roles in directing host responses to implants.^17,18^ In this study, we did not examine the effects of alloy degradation products on these pathways at the molecular level, and thus, further investigations are required in this direction.

Regarding the experimental part of our study, we used 3D µCT as well as 2D histological, immunohistochemical, histomorphometrical, and SAXS analyses to provide a broader overview of the alloy degradation behavior and early host responses to it. The data obtained from all analyses were in the same line and supported our hypothesis regarding the comparability of results obtained from 2 and 3D interface imaging technologies. However, we did not use in vivo µCT, which could provide better information about the H_2_ gas evolution, and did not consider the earlier time points (such as few hours after implantation). Consequently, we could not draw any conclusions about the gas formation at the interface. Because degradable materials interact chemically with the body, it would be more beneficial to develop chemical techniques to investigate the biochemical features of surface biocompatibility after implantation.^97^ Furthermore, we did not consider an inert titanium group as control group. However, we believed that owing to the degradability and chemical interactions between Mg surface alloys and the body, their influences on the immune system could not be compared to titanium implants as bio-inert materials.

## Conclusions

Biodegradable alloys made from Mg, Zn, and Ca, known as ZX alloys, have a potential in stimulating pediatric bone healing. In this study, we evaluated the early host responses to Mg 0.45wt%Zn-0.45wt%Ca pin-shaped alloy (known as ZX00 alloy) in juvenile rat animals 2, 5, and 10 days after implantation. Our results indicated that the ZX00 alloy could significantly stimulate macrophage polarization at the implant-bone interface 5 and 10 days after surgery. The activity of (ALP and Runx 2 biological markers reduced significantly for Mg group, demonstrating less osteoblast activity). However, after 10 days of implantation, we observed an insignificant improvement of the osteoblast activity (ALP and Runx 2) as well as collagen fibrils alignment and collagen/hydroxyapatite (HAp) size, compared to day 5 and sham group at all time. Taken together, our results supported that the ZX00 alloy could stimulate the expression of pro-healing type 2 macrophages phenotype in vivo; however, our biomineralization data did not represent any statistically significant differences over time.

## Supplemental Material

sj-docx-1-tej-10.1177_20417314211047100 – Supplemental material for Early osteoimmunomodulatory effects of magnesium–calcium–zinc alloys Click here for additional data file. Supplemental material, sj-docx-1-tej-10.1177_20417314211047100 for Early osteoimmunomodulatory effects of magnesium–calcium–zinc alloys by Maryam Rahmati, Sabine Stötzel, Thaqif El Khassawna, Kamila Iskhahova, DC Florian Wieland, Berit Zeller Plumhoff and Håvard Jostein Haugen in Journal of Tissue Engineering
