# Supplementary material for: Early osteoimmunomodulatory effects of magnesium–calcium–zinc alloys
Source: J Tissue Eng. 2021 Sep 22;12:20417314211047100. doi: 10.1177/20417314211047100 (PMC8474317; doi:10.1177/20417314211047100)
Supplement: sj-docx-1-tej-10.1177_20417314211047100 – Supplemental material for Early osteoimmunomodulatory effects of magnesium–calcium–zinc alloys [file sj-docx-1-tej-10.1177_20417314211047100.docx]

Supporting Information

***Early Osteoimmunomodulatory Effects of Magnesium–Zinc–Calcium alloys***

*Maryam Rahmati^1^, Sabine Stötzel^2^, Thaqif El Khassawna^2,3^, Kamila Iskhahova^4^, D.C. Florian Wieland^4^, Berit Zeller Plumhoff^4^, Håvard Jostein Haugen^1*^*

*^1^Department of Biomaterials, Institute for Clinical Dentistry, University of Oslo, Oslo, Norway*

*^2^Experimental Trauma Surgery, Justus-Liebig University Giessen, Giessen, Germany*

*^3^Faculty of Health Sciences, University of Applied Sciences, Giessen, Germany*

*^4^IInstitute of Metallic Biomaterials, Helmholtz Zentrum Hereon, Max-Planck-Straße 1, 21502, Geesthacht, Germany*

******Correspondence to:*** *Håvard Jostein Haugen*

*Email address:* [*h.j.haugen@odont.uio.no*](mailto:h.j.haugen@odont.uio.no)


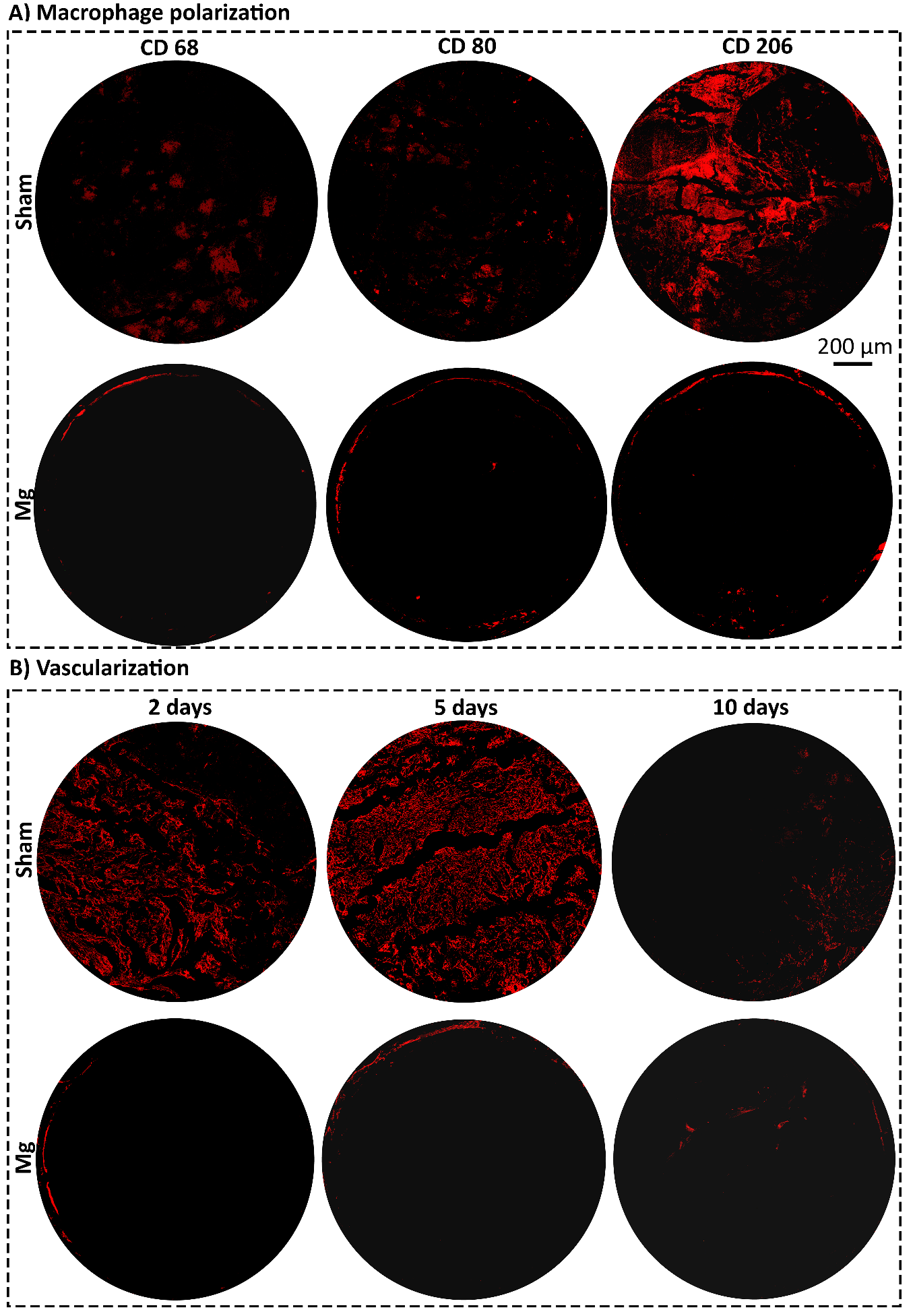


**Figure S1.** Fluorescent images of macrophage polarization and vascularization immunohistochemical analysis. A) Representative fluorescent images of antibody staining against CD68, 80 and 206 macrophage markers 10 days after implantation. B) Representative fluorescent images of alpha smooth muscle and Actin (α-SMA) staining for blood vessel formation in Mg-based alloy and sham groups over time, scale bar=200 µm.


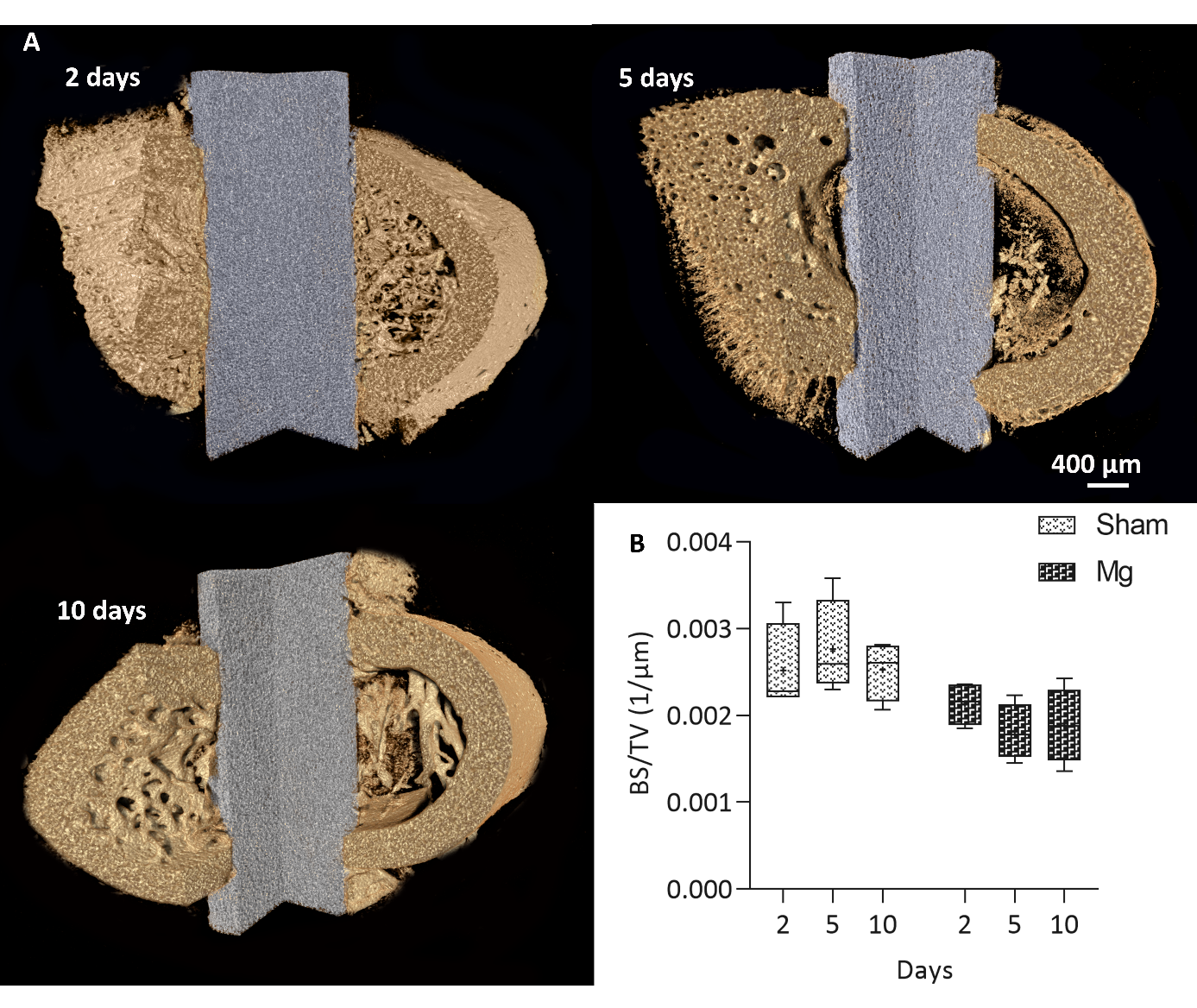


**Figure S2.** A) Representative wedge images of 3D evaluation of bone mineralization in the Mg based alloy group over time in the transverse plane, scale bar=500 µm. B) Quantitative data of bone surface to tissue volume (BV/TV) values over time using 3D µCT.
